# Multilevel Disparities of Sex-Differentiated Human Papilloma Virus-Positive Oropharyngeal Cancers in the United States

**DOI:** 10.3390/jcm13216392

**Published:** 2024-10-25

**Authors:** Rhea Verma, David J. Fei-Zhang, Lily B. Fletcher, Sydney A. Fleishman, Daniel C. Chelius, Anthony M. Sheyn, Jeffrey C. Rastatter, Jill N. D’Souza

**Affiliations:** 1 Northwestern University Feinberg School of Medicine, 420 E Superior St, Chicago, IL 60611, USA; 2 Department of Otolaryngology-Head and Neck Surgery, University of Tennessee Health Science Center, 910 Madison Avenue, Memphis, TN 38163, USA; 3 Department of Otolaryngology-Head and Neck Surgery, Pediatric Thyroid Tumor Program and Pediatric Head and Neck Tumor Program, Baylor College of Medicine, 6701 Fannin Street, Houston, TX 77030, USA; 4 Department of Pediatric Otolaryngology, Le Bonheur Children’s Hospital, Memphis, TN 38163, USA; 5 Department of Pediatric Otolaryngology, St. Jude Children’s Research Hospital, Memphis, TN 38105, USA; 6 Department of Otolaryngology-Head and Neck Surgery, Northwestern University Feinberg School of Medicine, 675 N Saint Clair, Chicago, IL 60611, USA; 7 Division of Pediatric Otolaryngology, Ann & Robert H. Lurie Children’s Hospital of Chicago, 225 East Chicago Avenue, Chicago, IL 60611, USA; 8 Department of Otolaryngology, Louisiana State University Health Sciences Center, 533 Bolivar St, New Orleans, LA 77012, USA; 9 Division of Pediatric Otolaryngology, Children’s Hospital of New Orleans, 200 Henry Clay Ave., New Orleans, LA 70118, USA

**Keywords:** social determinants, Yost Index, HPV, oropharyngeal cancer, gender, sex

## Abstract

**Objectives**: This study used multilevel social determinants of health (SDoH) models to determine how SDoH influence different sexes of patients diagnosed with HPV-positive oropharyngeal squamous cell cancers (OPSCC) across the US. **Methods**: This was a retrospective cohort study assessing HPV-confirmed patients with oropharyngeal squamous cell cancers from 2010 to 2018 using census-level Yost Index socioeconomic status (SES) score and rurality–urbanicity measures alongside individual-level race–ethnicity while stratifying by biological sex. Age-adjusted multivariate regressions were performed for survival, treatment receipt, and delay of treatment initiation (of 3+ months). **Results**: Across 14,076 OPSCC-HPV-positive patients, delay of treatment uniquely featured positive predictors for males of black race–ethnicity (OR, 2.07; 95% CI, 1.68–2.54) and poor Yost SES (1.43; 1.24–1.65). Five-year all-cause mortality uniquely showed positive predictors of females of black race–ethnicity (2.74; 1.84–4.71) and of males with poor Yost SES (1.98; 1.79–2.19). Three-year all-cause mortality shared positive predictors across sexes but were exacerbated in females of black race–ethnicity (2.50; 1.82–3.44) compared to males (2.23; 1.91–2.60); this was reversed for poor Yost SES (male, 1.92, 1.76–2.10; female, 1.60, 1.32–1.95). Surgery showed negative predictors of black race–ethnicity that displayed worsened effects in females (0.60, 0.44–0.79) versus males (0.75, 0.66–0.86). First-line radiation receipt uniquely featured negative predictors for males of black race–ethnicity (0.73; 0.62–0.86) with poor Yost SES (0.74; 0.68–0.82). **Conclusions**: Comprehensive models of multilevel SDoH displayed exacerbated disparity effects of community-level SES in males and black race–ethnicity among female HPV-positive OPSCC patients. These objective comparisons of specific SDoH factors inform providers and policy direction on how to strategically target the most pertinent SDoH factors affecting a rapidly growing cancer population.

## 1. Introduction

Human papillomavirus (HPV) infection has emerged as a significant risk factor for oropharyngeal squamous cell carcinoma (OPSCC), with approximately 80% of OPSCC cases in the United States associated with HPV-infection [1,2,3,4]. Patients diagnosed with HPV-positive (+) OPSCC often exhibit characteristics such as younger age, better overall health, and higher socioeconomic status (SES) compared to those with HPV-negative (−) disease [5,6]. Notably, oropharyngeal cancer now surpasses cervical cancer as the most prevalent HPV-related cancer in the United States, further emphasizing the clinical significant of HPV(+) status in oncology [7].

Since 2010, the National Cancer Institute Surveillance, Epidemiology, and End Results (SEER) has collected data on the oral HPV status, socioeconomic information, as well as details of patient cancer stage, treatment, survival, etc. Retrospective studies using the SEER database have highlighted that social determinants of health have been associated with OPSCC prognosis [3,8,9]. Many studies have shown inferior outcomes for OPSCC patients of minoritized race–ethnic and low-SES backgrounds [3,6,10]. Factors such as higher stage at diagnosis, comorbidities, access to care, and treatment differences have also been studied as contributing to survival disparities [11,12,13]. 

The relevance of biological sex as an SDoH and its role in health inequities is now broadly acknowledged [14,15,16,17,18]. However, its role in HPV(+) OPSCC etiology is not well established, despite its growing presence across both biological sexes due to increasing patterns of oral-sexual behaviors [19,20,21,22]. Furthermore, within HPV(+) OPSCC, biological sex has not been a primary focus when contextualizing the impact of a wide variety of SDoH factors. Thus, our study aims to utilize a specialized SEER database tailored for examining oropharyngeal cancers with HPV-confirmed statuses and their associations with a variety of community-level and individual-level SDoH factors across male and female patients. We hypothesized that community-level SDoH factors, namely socioeconomic status factors, will showcase similar-magnitude associations with different mortality and treatment outcomes when comparing both sex cohorts while individual-level SDoH factors, namely race–ethnicity, will have different-magnitude associations between sex cohorts.

## 2. Methods

This retrospective cohort study followed the Strengthening the Reporting of Observational Studies in Epidemiology (STROBE) reporting guidelines. In using retrospective, deidentified public databases, the Institutional Review Board of Northwestern University has exempted this from the approval process per their policies. 

### 2.1. Databases

The SEER database utilized for this analysis was the specially authorized incidence database for head and neck tumors with linked census tract-level socioeconomic status (SES), rurality–urbanicity, as well as confirmed HPV status spanning patients from 2010 to 2018 and 18 registries across the country.

The Yost Index is a validated, composite SDoH measure provided within this specialized dataset and comprises the following seven census-level measures spanning all 72,158 US Census tracts: median household income, percentage of individuals below 150% of poverty line, median house value, median rent, percentage working-class employment, percentage unemployment, and education index level. More details regarding this index’s incorporation can be found in the Supplementary Materials.

Rurality–urbanicity is provided in the specialized SEER database and comprises two measures. The first is the US Department of Agriculture’s Rural Urban Commuting Area (RUCA). RUCA is delineated by “Urban” and “Not Urban” and is assigned on a per-individual basis. The second is the Urban Rural Indicator Code (URIC) based on the US Census summary measure of “percentage of a population living in non-urban areas” in the 5-year estimates. These are indicated by four categories of “all urban”, “mostly urban”, “mostly rural”, and “all rural” and are also assigned on a per-individual basis. Similar to Yost Index measures, in order to preserve the deidentified status of individual patients, specific geographies are not revealed by the SEER database.

### 2.2. Population Definitions

Oropharyngeal squamous cell carcinomas (OPSCC) were extracted based on International Classification of Disease for Oncology version 3.8 (ICD-O-3) tumor site codes [C10.0–10.9] and diagnosis between 2010 and 2018. Histological classifications of squamous cell carcinoma were determined by ICD-O-3 codes re-categorized by SEER-determined labels. Inclusion criteria were adult patients (20+ years) with confirmed HPV-positive status, which was determined by IHC for p16 expression as per SEER record administrators. Further details regarding HPV-confirmed status can be found within SEER documentation. 

### 2.3. Characteristics and Outcome Variable Definitions

Individual-level sociodemographic/SDoH variables consisted of age (i.e., <65 and 65+ years as the categories), gender/biological sex, race–ethnicity (non-Hispanic white, black, Hispanic, Asian, and Native Pacific Islander) on a per-patient basis.

Community-level SDoH variables consisted of census-level Yost Index SES score and a composite urbanicity–rurality measure comprised of the two available SEER variables. For the former, patients were separated into two equivalently sized cohorts of “Lower SES”, which encompassed quintiles of “Lowest” and “Lower”, and “Middle” SES, which comprised quintiles of “Higher” and “Highest” SES. For the latter, rurality status was determined when the patient either had a RUCA designation of “Non-Urban” or a URIC designation of “Mostly Rural” or “All Rural”.

Outcome variables consisted of the following: all-cause-related mortality was determined by SEER-categorized variables, alongside time points for three-year and five-year mortality.

Advanced staging for malignant tumors was based on SEER-designated variables labeled as “Stage IV”, “distant [expansion]”, or “distal [expansion]” and recoded under American Joint Committee on Cancer, 6th Edition (AJCC-6) classifications. Delay in treatment after diagnosis was defined as treatment beginning 3 or more months after preliminary diagnosis.

### 2.4. Statistical Analysis

All multivariate models consisted of the following: covariates of being of younger age/<65 years (reference being 65+ years), being of black race–ethnicity (reference not being of black race–ethnicity), being of Hispanic race–ethnicity (reference not being of Hispanic race–ethnicity), being of Asian or Pacific Islander race–ethnicity (reference not being of Asian or Pacific Islander race–ethnicity), Yost Index/”Lower Socioeconomic Status” (being in the Lowest, Lower, or Middle SES quintiles, reference being in the Higher, Highest SES quintiles), and increased rurality (being of “rural” RUCA-designation, “all rural” or “mostly rural” URIC-designations; reference being “urban” RUCA-designation, “all urban” or “mostly urban” URIC-designations), stratifying the variate of being either male or female sex.

Multivariate cox-proportional hazards models (i.e., Hazard Ratios) were utilized to assess overall time to all-cause mortality. This was utilized to assess events of confirmed mortality while censoring significant events that do not portray a confirmed alive or dead status within SEER.

Multivariate logistic regression models (i.e., Odds Ratios) were also used to delineate covariate effects on aspects of mortality not well represented within cox-proportional models of overall mortality/survival, which principally focused on the whole timespan of mortal events with time as a continuous covariate while censoring significant events that obscured covariate effects at specific time points. More details behind this approach’s rationale can be found in the Supplementary Materials.

Multivariate logistic regression models were also used to assess the odds of having a late-presenting stage (based on SEER-recoded combined staging, reference level being diagnosed with Stage I–III).

These regressions were performed as stratified, independent multivariate models for each male and female sex groups. Two-sided *p*-values were calculated, and *p* < 0.05 was set as the alpha-criterion for statistical significance. All statistical analyses were performed using R-version 4.2.1.

## 3. Results

### 3.1. Patient Demographics

A total of 14,076 HPV-positive OPSCC patients were identified, of which 1877 (13%) were female, and 12,199 (87%) were male. Amongst patients of female sex, the highest representing sociodemographic characteristics were ages 45–64 (n = 1063, 57%) and white race (n = 1529, 81%). Amongst male identifying patients, ages 45–64 (n = 10,434, 86%) and white race (n = 10,434, 86%) had the most representation. The highest represented census-level Yost Socioeconomic Status Index Score for both female (n = 923, 53%) and male (n = 6673, 58%) patients was High SES. Both female (n = 1164, 64%) and male (n = 7378, 63%) patients were mostly representative of “All Urban” rural–urbanicity. The highest represented clinical characteristics are Stage IV and Above per AJCC-6 overall TNM classifications in females (n = 1192, 64%) and males (n = 9096, 75%). These characteristics and their chi-squared analysis significance are fully displayed in Table 1.

### 3.2. Mortality

Mortality risk differences across sex cohorts were assessed across three outcomes: overall all-cause mortality, 3-year all-cause mortality, and 5-year all-cause mortality for HPV(+) oropharyngeal cancer patients.

#### 3.2.1. Overall All-Cause Mortality

In both male and female (HPV)+ OPSCC patients, an increased overall all-cause mortality with being of black race and having lower Yost Index SES was noted. However, males showed markedly greater effects for lower Yost Index SES (male, HR 1.70; 95% CI 1.59–1.81|female, HR 1.41, 95% CI 1.23–1.61), while females showed minimally increased effects for being of black race (males, HR 1.78; 95% CI 1.61–1.96|females, HR 1.88; 95% CI 1.55–2.28) (*p* < 0.001 all).

Both sex cohorts also displayed similarly decreased magnitudes of overall mortality risk with younger age (<65 years) (male, HR 0.49; 95% CI 0.46–0.52; female, HR 0.51; 95% CI 0.45–0.58) (*p* < 0.001 all) (Table 2).

#### 3.2.2. 3-Year All-Cause Mortality

In both male and female patients, increased odds of 3-year all-cause mortality were observed with black race–ethnicity and lower Yost Index SES. However, males experienced stronger effects with lower Yost Index SES (male, OR 1.92, 95% CI 1.76–2.10, *p* < 0.001|females, OR 1.60, 95% CI 1.32–1.95, *p* < 0.001), while females experienced minimally stronger effects with being of black race–ethnicity (males, OR 2.23, 95% CI 1.91–2.6, *p* < 0.001|females, OR 2.50, 95% CI 1.82–3.44, *p* < 0.001).

For male patients only, being of Hispanic race–ethnicity showed increased 3-year all-cause mortality (OR 1.30, 95% CI 1.09–1.54, *p* = 0.004).

In both male and female HPV(+) cancers, younger age showed similarly decreased odds of 3-year all-cause mortality (respectively OR 0.41, 95% CI 0.37–0.44, *p* < 0.001 and OR 0.43, 95% CI 0.36–0.51, *p* < 0.001) (Table 3).

#### 3.2.3. Five-Year All-Cause Mortality

Both male and female patients showed increased odds of 5-year all-cause mortality if they were of black race–ethnicity and lower Yost Index SES. However, males displayed markedly stronger effects with lower Yost Index SES (male, OR 1.98, 95% CI 1.79–2.19, *p* < 0.001|female, OR 1.57, 95% CI 1.26–1.96, *p* < 0.001), while females displayed minimally stronger effects with being of black race–ethnicity (male, OR 2.41, 95% CI 1.99–2.94, *p* < 0.001|female, OR 2.74, 95% CI 1.84–4.17, *p* < 0.001)

For male patients only, being of Hispanic race–ethnicity showed increased odds of 5-year all-cause mortality (OR 1.23, 95% CI 1.01–1.50, *p* = 0.045). For female patients only, increasing the rurality of their community showed decreased odds of 5-year mortality (OR 0.64, 95% CI 0.43–0.97, *p* = 0.035) but not for male patients.

In both male and female patients, younger age showed similarly decreased odds of 5-year all-cause mortality (male, OR 0.32, 95% CI 0.29–036, *p* < 0.001|female, OR 0.37, 95% CI 0.29–0.45, *p* < 0.001) (Table 3).

### 3.3. Staging

For both male and female HPV(+) OPSCC patients, increased odds of having an advanced stage on preliminary presentation were observed for younger aged patients, with stronger effects observed among males (male, OR 1.37, 95% CI 1.27–1.47, *p* < 0.001|females, OR 1.19, 95% CI 1.02–1.38, *p* = 0.027).

Race–ethnicity, Yost index SES, and rural–urbanicity showed significant independent influences on staging when analyzed in these multivariate models (Table 4).

### 3.4. Treatment

Multivariate analyses were conducted regarding three treatment options for oropharyngeal HPV(+) cancers: surgery, radiation therapy, and chemotherapy, as well as overall delay of treatment initiation.

#### 3.4.1. Delay of Treatment Initiation

For both male and female HPV(+) OPSCC patients, being of black race showed increased odds of having a delay of treatment initiation after primary diagnosis, with male patients experiencing markedly stronger effects (male, OR 2.07; 95% CI 1.6–2.54; *p* < 0.001|female, OR 1.69; 95% CI 1.06–2.61; *p* = 0.028).

For male patients only, being of Hispanic race–ethnicity (OR 1.48; 95% CI 1.15–1.89; *p* = 0.003) and having lower Yost Index SES (OR 1.43; 95% CI 1.24–1.65; *p* < 0.001) showed markedly increased odds of having delay of treatment initiation. In addition, male patients living in rural communities showed markedly decreased odds of having a delay of treatment initiation (OR 0.63; 95% CI 0.45–0.86; *p* = 0.003) (Table 5).

#### 3.4.2. Primary Surgery Receipt

When analyzing both male and female HPV(+) cancer patients, younger age (male, OR 1.43, 95% CI 1.34–1.54, *p* < 0.001|female, OR 1.41, 95% CI 1.21–1.65, *p* < 0.001) showed similarly increased odds of receiving surgery. For both cohorts, being of black race showed minimally decreased odds of surgery receipt that was exacerbated among female patients (male, OR 0.75; 95% CI 0.66–0.86, *p* < 0.001|female, OR 0.60; 95% CI 0.44–0.79, *p* < 0.001).

For male patients only, lower Yost Index SES showed decreased odds (OR 0.83, 95% CI 0.78–0.89, *p* < 0.001) of surgical treatment, while rurality showed increased odds (OR 1.18, 95% CI 1.03–1.35, *p* = 0.017) of surgical treatment (Table 6).

#### 3.4.3. Radiation Therapy Receipt

Amongst both male and female HPV(+) OPSCC patients, younger age showed increased odds of receiving radiation therapy, with somewhat stronger magnitudes observed within female patients (male, OR 1.54, 95% CI 1.40–1.69, *p* < 0.001|female, OR 1.66, 95% CI 1.39–1.99, *p* < 0.001). For both cohorts, lower Yost Index SES showed decreased odds of radiation therapy receipt, with stronger associations observed among male patients (male, 0.74, 0.68–0.82, *p* < 0.001|female, 0.80, 0.66–0.97, *p* = 0.020).

For male patients only, being of black race demonstrated decreased odds of radiation therapy treatment (OR 0.73, 95% CI 0.62–0.86, *p* < 0.001). For female patients only, rurality (OR 1.55, 95% CI 1.04–2.39, *p* = 0.031) demonstrated increased odds of radiation therapy (Table 6).

#### 3.4.4. Chemotherapy Receipt

Amongst both male and female HPV(+) OPSCC patients, younger age showed markedly increased odds of receiving chemotherapy (male, OR 1.70, 95% CI 1.58–1.83, *p* < 0.001|female, OR 1.70, 95% CI 1.46–1.98, *p* < 0.001).

For male patients only, being of black race displayed decreased odds of receiving chemotherapy (OR 0.87, 95% CI 0.76–1.00, *p* = 0.046). For female patients only, being of Asian or Pacific Islander race–ethnicity demonstrated markedly decreased odds of chemotherapy receipt (OR 0.64, 95% CI 0.44–0.94, *p* = 0.022) but markedly increased odds with rurality (OR 1.60, 95% CI 1.16–2.24, *p* = 0.004) (Table 6).

## 4. Discussion

To our knowledge, this is the first study to show independent associations of a wide variety of community- and individual-level SDoH factors with disparities of HPV(+) oropharyngeal squamous cell carcinomas differentiated by biological sex/gender. For all-cause mortality disparities, community-level Yost Index SES and individual-level black race–ethnicity largely impacted both cohorts. However, community-level SES effects were exacerbated among males, while black race–ethnicity effects were exacerbated among females. For treatment-level disparities, community- and individual-level factors showed differing magnitudes of associations across analyses of outcomes stratified by biological sex.

Across earlier and longer-term mortality observations, lower community-level SES was detrimentally associated with both sexes diagnosed with HPV(+) OPSCC, with exacerbated effects experienced among males. Although the significance of SES affecting HPV-associated OPSCC has long been accepted, our observations of differing magnitudes across sexes provides nuanced context for how specific HPV-OPSCC populations experience disparities [3,23,24]. When considering our observations of community-level SES solely affecting male patients in receiving less surgery and having increased delays of treatment initiation, our mortality and treatment results in tandem showcase how treatment disparity mechanisms impart stronger prognostic inequities among males, with HPV(+) OPSCC males hinging on community-level SES. Though such evidence purporting the linkage between SES, treatment disparity, and prognostic outcomes has been highlighted for HPV(+) OPSCC in general [2,8,25], this present study provides principal insights into how observations of its worsened population-level effects may predominantly lie within the male patient population.

In addition to this engendered quality of community-level SES detriment, individual-level black race–ethnicity showed detrimental effects across both sexes but displayed exacerbated influence among female patients with HPV(+) OPSCC. Similar to SES, being of black race–ethnicity, among other minoritized groups, has been observed for its associations with HPV(+) OPSCC prognostic and treatment disparities compared to white race–ethnic patient groups [3,26,27]. Our present investigation furthers these findings by contextualizing how this disparity may be more strongly experienced by female patients. However, unlike our observations of community-level SES and the linkage between treatment and prognosis, our results do not clearly delineate a similar gendered treatment disparity mechanism for being of black race–ethnicity leading to worsened mortality differences. It should be noted, however, that being of black race–ethnicity did showcase decreased radiation therapy receipt for both male and female patients. Given that other explicative community- and individual-level factors had imparted treatment disparities, including rurality–urbanicity for radiation therapy and being of Asian-Pacific Islander race–ethnicity for chemotherapy, these results infer how patients who are not of black race–ethnicity are likely experiencing multifaceted SDoH influences to impart the population-level disparities observed for HPV(+) OPSCC patients. Given that such factors for HPV-confirmed OPSCC have largely been under investigated due to a dearth of patient data, future investigations should look towards building more diverse patient datasets in characterizing how these SDoH characteristics would impart HPV-OPSCC-related disparities, especially among different sexes. In addition, clinical mechanisms beyond the ones observed in our study may be responsible for these population-level disparities, such as health literacy and long-term follow-up care adherence, as has been previously hypothesized [2,8,25,26,27,28,29,30,31]. Along those lines, these aspects of literacy, education, and long-term surveillance should be further explored within the contexts of HPV infection status, which has long been associated with younger patients and sociobehavioral differences across differing population subgroups [2,8,25].

This study must be considered within its limitations. Although specially designed for studying multilevel social determinant factors pertaining to HPV-related head–neck cancers, the SEER database utilized in this study does not encompass the entirety of variables that would be of investigative interest. These include further clinical variables such as the depth of primary invasion, hospital policies for rural vs urban locations, or specific treatment details beyond the general descriptions of surgery, chemotherapy, or radiation therapy receipt. Despite being one of the larger cohort studies focusing solely on HPV(+) OPSCC, there is a notable difference in sample size between gender status groups, reflecting the male predominance of other OPSCC studies [32,33,34,35]. However, such weaknesses were considered for by incorporating a large absolute sample of female patients (n = 1877) diagnosed within the past decade, the utilization of statistical methods (multivariate logistical regressions and cox-proportional hazards) that proportionally widened the confidence interval when dealing with disparate sample sizes, and the stratified approach of independent, sex cohort models.

## 5. Conclusions

This investigation into HPV-associated oropharyngeal squamous cell cancers not only expands upon prior investigations regarding socioeconomic status and race–ethnicity impact on all-cause patient mortality, delay in treatment, and primary treatment modality but also sheds light on how the intricate associations between multilevel sociodemographic factors imparts disparate outcomes differentiated by biological sex/gender. By observing a variety of outcomes across mortality, staging, type of treatment received, and timing of treatment initiation, our findings showcase the multifaceted associations of individual- and community-level SDoH factors as to how they universally and uniquely affect disparities of male and female patients with HPV(+) OPSCC. In turn, these allow targeted interventions to be proposed and initiated with particular HPV-related OPSCC patients that demonstrate the most need in healthcare access and delivery.

## Figures and Tables

**Table 1:** Clinicodemographic characteristics by biological sex.

		Sex		
Characteristic	n	Female, n = 1877 (13%)	Male, n = 12,199 (87%)	*p*-Value
**Age**	14,076			<0.001
20–44 years		106 (5.6%)	443 (3.6%)	
45–64 years		1063 (57%)	7830 (64%)	
65–84 years		667 (36%)	3790 (31%)	
85+ years		41 (2.2%)	136 (1.1%)	
**Race**	14,076			<0.001
White		1529 (81%)	10,434 (86%)	
Hispanic		147 (7.8%)	730 (6.0%)	
Black		118 (6.3%)	650 (5.3%)	
Asian or Pacific Islander		66 (3.5%)	278 (2.3%)	
Native American		11 (0.6%)	54 (0.4%)	
Unknown		6 (0.3%)	53 (0.4%)	
**Yost Socioeconomic Status Index Score (US)**	13,295			<0.001
High SES		923 (52%)	6673 (58%)	
Low-Middle SES		850 (48%)	4849 (42%)	
**US Census Rural–Urbanicity**	13,546			0.342
All Rural		125 (6.9%)	761 (6.5%)	
All Urban		1164 (64%)	7378 (63%)	
Mostly Rural		135 (7.5%)	903 (7.7%)	
Mostly Urban		383 (21%)	2697 (23%)	
**AJCC-TNM Staging**	13,970			<0.001
Stage I-III		670 (36%)	3012 (25%)	
Stage IV & Above		1192 (64%)	9096 (75%)	
**No. of Primary Tumors by Dx**	14,076			<0.001
1		1417 (75%)	9752 (80%)	
2 or more		460 (25%)	2447 (20%)	
**Primary Surgery Performed**	13,977			<0.001
No Surgery		1067 (57%)	7725 (64%)	
Surgery		798 (43%)	4387 (36%)	
**Radiation Therapy Performed**	14,076			<0.001
No Therapy		286 (15%)	1502 (12%)	
Therapy		1591 (85%)	10,697 (88%)	
**Chemotherapy Performed**	14,076			<0.001
No Therapy		665 (35%)	3487 (29%)	
Therapy		1212 (65%)	8712 (71%)	
**Months from Diagnosis to Initial Treatment**	13,663			0.021
<1 month		827 (45%)	4972 (42%)	
1–3 months		962 (53%)	6651 (56%)	
3 months or more		36 (2.0%)	215 (1.8%)	
**3-year All-cause Mortality**	9020			>0.999
Alive, >3 yr		898 (75%)	5895 (75%)	
Dead, ≤3 yr		294 (25%)	1933 (25%)	
**5-year All-cause Mortality**	5901			0.226
Alive, >5 yr		450 (58%)	2828 (55%)	
Dead, ≤5 yr		331 (42%)	2292 (45%)	
**Vital Status on Last Follow-up**	14,076			0.188
Alive		1529 (81%)	9775 (80%)	
Dead		348 (19%)	2424 (20%)	

**Table 2:** Overall mortality stratified by biological sex.

		Male			Female		
Primary Site ^1^	Characteristic	HR	95% CI	*p*-Value	HR	95% CI	*p*-Value
**Oropharynx**	Younger Age (<65 years)	0.49	0.46, 0.52	<0.001	0.51	0.45, 0.58	<0.001
	Race—Black	1.78	1.61, 1.96	<0.001	1.88	1.55, 2.28	<0.001
	Race—Hispanic	1.14	1.00, 1.29	0.053	0.96	0.73, 1.27	0.793
	Race—Asian or Pacific Islander	1.17	0.96, 1.42	0.128	0.96	0.68, 1.35	0.807
	Yost Index—Lower Socioeconomic Status	1.70	1.59, 1.81	<0.001	1.41	1.23, 1.61	<0.001
	Rurality	1.00	0.88, 1.12	0.964	0.85	0.65, 1.12	0.236

^1^ By International Classification of Diseases for Oncology, 3rd edition (ICD-O-3).

**Table 3:** Three- and five-year mortality stratified by biological sex.

		Male			Female		
Mortality	Characteristic	OR	95% CI	*p*-Value	OR	95% CI	*p*-Value
**3-Year Mortality**	Younger Age (<65 years)	0.41	0.37, 0.44	<0.001	0.43	0.36, 0.51	<0.001
	Race—Black	2.23	1.91, 2.60	<0.001	2.50	1.82, 3.44	<0.001
	Race—Hispanic	1.30	1.09, 1.54	0.004	1.26	0.85, 1.85	0.251
	Race—Asian or Pacific Islander	1.17	0.89, 1.53	0.271	0.83	0.51, 1.34	0.452
	Yost Index—Lower Socioeconomic Status	1.92	1.76, 2.10	<0.001	1.60	1.32, 1.95	<0.001
	Rurality	0.99	0.84, 1.18	0.948	0.93	0.64, 1.35	0.717
**5-Year Mortality**	Younger Age (<65 years)	0.32	0.29, 0.36	<0.001	0.37	0.29, 0.45	<0.001
	Race—Black	2.41	1.99, 2.94	<0.001	2.74	1.84, 4.17	<0.001
	Race—Hispanic	1.23	1.00, 1.50	0.045	1.17	0.75, 1.84	0.486
	Race—Asian or Pacific Islander	1.34	0.97, 1.84	0.073	0.94	0.54, 1.64	0.830
	Yost Index—Lower Socioeconomic Status	1.98	1.79, 2.19	<0.001	1.57	1.26, 1.96	<0.001
	Rurality	1.04	0.85, 1.26	0.732	0.64	0.43, 0.97	0.035

**Table 4:** Advanced staging at presentation stratified by biological sex.

		Male			Female		
Primary Site ^1^	Characteristic	OR	95% CI	*p*-Value	OR	95% CI	*p*-Value
**Oropharynx**	Younger Age (<65 years)	1.37	1.27, 1.47	<0.001	1.19	1.02, 1.38	0.027
	Race—Black	1.11	0.97, 1.29	0.141	1.23	0.93, 1.64	0.151
	Race—Hispanic	1.09	0.94, 1.27	0.239	1.08	0.80, 1.47	0.598
	Race—Asian or Pacific Islander	1.26	0.99, 1.60	0.059	0.86	0.59, 1.25	0.419
	Yost Index—Lower Socioeconomic Status	1.02	0.94, 1.09	0.694	1.12	0.96, 1.31	0.158
	Rurality	0.99	0.85, 1.15	0.870	1.16	0.85, 1.60	0.354

^1^ By International Classification of Diseases for Oncology, 3rd edition (ICD-O-3).

**Table 5:** Delays in treatment stratified by biological sex.

		Male			Female		
Primary Site ^1^	Characteristic	OR	95% CI	*p*-Value	OR	95% CI	*p*-Value
**Oropharynx**	Younger Age (<65 years)	0.86	0.75, 0.99	0.040	0.88	0.66, 1.17	0.375
	Race—Black	2.07	1.68, 2.54	<0.001	1.69	1.06, 2.61	0.028
	Race—Hispanic	1.48	1.15, 1.89	0.003	1.37	0.79, 2.23	0.250
	Race—Asian or Pacific Islander	1.28	0.82, 1.89	0.261	0.65	0.23, 1.46	0.322
	Yost Index—Lower Socioeconomic Status	1.43	1.24, 1.65	<0.001	1.13	0.84, 1.52	0.434
	Rurality	0.63	0.45, 0.86	0.003	1.02	0.55, 1.76	0.957

^1^ By International Classification of Diseases for Oncology, 3rd edition (ICD-O-3).

**Table 6:** Multimodal treatment receipt stratified by biological sex.

		Male			Female		
Treatment Type	Characteristic	OR	95% CI	*p*-Value	OR	95% CI	*p*-Value
**Surgery**	Younger Age (<65 years)	1.43	1.34, 1.54	<0.001	1.41	1.21, 1.65	<0.001
	Race—Black	0.75	0.66, 0.86	<0.001	0.60	0.44, 0.79	<0.001
	Race—Hispanic	1.04	0.91, 1.19	0.583	1.11	0.82, 1.48	0.496
	Race—Asian or Pacific Islander	1.03	0.83, 1.27	0.804	0.98	0.67, 1.43	0.926
	Yost Index—Lower Socioeconomic Status	0.83	0.78, 0.89	<0.001	0.89	0.76, 1.04	0.148
	Rurality	1.18	1.03, 1.35	0.017	0.99	0.72, 1.34	0.944
**Radiation**	Younger Age (<65 years)	1.54	1.40, 1.69	<0.001	1.66	1.39, 1.99	<0.001
	Race—Black	0.73	0.62, 0.86	<0.001	0.88	0.65, 1.23	0.455
	Race—Hispanic	0.86	0.72, 1.03	0.094	0.90	0.64, 1.29	0.548
	Race—Asian or Pacific Islander	0.89	0.68, 1.20	0.449	0.81	0.53, 1.28	0.355
	Yost Index—Lower Socioeconomic Status	0.74	0.68, 0.82	<0.001	0.80	0.66, 0.97	0.020
	Rurality	1.17	0.97, 1.42	0.097	1.55	1.04, 2.39	0.031
**Chemotherapy**	Younger Age (<65 years)	1.70	1.58, 1.83	<0.001	1.70	1.46, 1.98	<0.001
	Race—Black	0.87	0.76, 1.00	0.046	1.10	0.84, 1.46	0.489
	Race—Hispanic	0.94	0.82, 1.08	0.392	0.82	0.62, 1.11	0.194
	Race—Asian or Pacific Islander	1.09	0.87, 1.37	0.472	0.64	0.44, 0.94	0.022
	Yost Index—Lower Socioeconomic Status	1.00	0.93, 1.07	0.968	0.86	0.73, 1.00	0.053
	Rurality	0.96	0.83, 1.11	0.575	1.60	1.16, 2.24	0.004

## Data Availability

This data is publicly available through the NCI-SEER, CDC, and FDA websites.

## Supplementary Materials

The following supporting information can be downloaded at: https://www.mdpi.com/article/10.3390/jcm13216392/s1, Table S1: Multivariate Logistic Regression Hosmer-Lemeshow Goodness of Fit Tests.
