# Supplementary material for: Multilevel Disparities of Sex-Differentiated Human Papilloma Virus-Positive Oropharyngeal Cancers in the United States
Source: J Clin Med. 2024 Oct 25;13(21):6392. doi: 10.3390/jcm13216392 (PMC11546109; doi:10.3390/jcm13216392)
Supplement: Supplementary file 1 [file jcm-13-06392-s001.zip › jcm-3259272-supplementary.pdf]

Supplement Table S1. Multivariate Logistic Regression Hosmer-Lemeshow Goodness of Fit Tests

Logistic Regression Model by Outcome	P-value[1]
**Advanced Staging**	
Male	0.892
Female	0.532
**Surgery Receipt**	
Male	0.398
Female	0.325
**Radiation Therapy Receipt**	
Male	0.388
Female	0.736
**Chemotherapy Receipt**	
Male	0.357
Female	0.904
**Delay in Treatment**	
Male	0.381
Female	0.335
**3-year Mortality**	
Male	0.143
Female	0.264
**5-year Mortality**	
Male	0.060
Female	0.051

1. P-value for Goodness of Fit Hosmer-Lemeshow. $P > 0.05$ indicates the model is a good fit
